# The relationship between organizational climate and job satisfaction of kindergarten teachers: a chain mediation model of occupational stress and emotional labor

**DOI:** 10.3389/fpsyg.2024.1373892

**Published:** 2024-05-28

**Authors:** Wei Xia, Yuchen Fan, Jingyu Bai, Qingyi Zhang, Yue Wen

**Affiliations:** School of Preschool and Elementary Education, China West Normal University, Nanchong, China

**Keywords:** kindergarten, organizational climate, kindergarten teachers, occupational stress, emotional labor, job satisfaction

## Abstract

Organizational climate has been shown to be an important factor associated with teachers’ job satisfaction. However, the internal mechanism between them is unclear. The purpose of this study was to investigate whether the relationship between kindergarten organizational climate and kindergarten teachers’ job satisfaction was affected by occupational stress and emotional labor. This study employed a questionnaire survey method to gather data from 1,091 kindergarten teachers nationwide. It conducted an analysis of the current status of kindergarten organizational climate and the job satisfaction of kindergarten teachers, elucidating the relationship between the two and the underlying mechanisms. Additionally, a chain mediation model was constructed. The findings indicated that: (1) organizational climate, kindergarten teachers’ occupational stress and emotional labor all significantly predict kindergarten teachers’ job satisfaction directly (2) organizational climate could indirectly influence kindergarten teachers’ job satisfaction through three pathways: the separate mediating effect of occupational stress and emotional labor, and the chain mediating effect on both. The research findings highlight the significance of kindergarten organizational climate, occupational stress, and emotional labor in augmenting the job satisfaction of kindergarten teachers, offering valuable insights for the improvement of kindergarten teacher job satisfaction.

## 1 Introduction

Teachers constitute a crucial pillar in attaining balanced and high-quality education. As essential contributors to the teaching workforce, kindergarten teachers assume a pivotal role in influencing the high-quality development of early childhood education. Teacher job satisfaction refers to the positive and contented state in which teachers express satisfaction with their own work (Judge and Kammeyer-Mueller, 2012), it is an overall and emotionally charged psychological experience and subjective evaluation made by teachers regarding the nature, content, conditions, and environmental aspects of their profession (Yao et al., 2016). Teachers’ job satisfaction inquiry and analysis can help managers not only comprehend teachers’ professional attitudes and avoid burnout but also provide some guidance for management decision-making (Hoque et al., 2023). The job satisfaction of kindergarten teachers not only affects the quality of teaching and learning (Yang et al., 2022), but it may also influence the overall cohesion of the kindergarten (Toropova et al., 2021). Ultimately, kindergarten teachers who are satisfied with their work can have a more positive impact on young children (Shoshani and Eldor, 2016), facilitating their comprehensive and harmonious physical and mental development. Consequently, this study is committed to investigating the job satisfaction of kindergarten teachers, unraveling the factors and mechanisms that influence it, with substantial empirical implications for enhancing the quality of early childhood education.

### 1.1 Organizational climate and job satisfaction

The organizational climate mirrors the culture, values, and working climate within an institution. In the context of schools, these factors are particularly reflected in how teachers collectively perceive and assess their work (Shi and Gheng, 2021). It involves shared perceptions of the practices and procedures encountered by teachers, as well as the behaviors they witness to attain rewards, support, expectations, and other elements (Schneider et al., 2013). Its core focuses on the description of how organizational influences impact organizational members (Ghavifekr and Pillai, 2016). These organizational factors include communication styles, culture, industry background, organizational structure, group dynamics, and leadership style (Banwo et al., 2022). A positive working environment can enhance employee loyalty, and studies have shown that excellent leaders are able to inspire employees to exert an additional 20–30% effort, leading to different performance outcomes (Hua, 2022). The ability of the organizational climate to attract employees directly influences their job satisfaction and satisfaction level with the job. According to the two-factor theory, people’s job satisfaction is often closely tied to factors like company policies, interpersonal relationships, and the work environment. Additionally, individuals’ emotional satisfaction with work, including a sense of achievement, rewards, and expectations for future development, also contributes to the shaping of the organizational climate (Shen, 2022). Hoppock (1953) observed that employees’ job satisfaction is influenced by individual physiological, psychological, and environmental factors, shaping employees’ feelings about their work (Mayo, 1933). Robbins and Judge (2017) propose that job satisfaction is a positive attitude toward work, and when job satisfaction is high, employees tend to have a positive attitude toward their work. School managers, armed with an understanding of this progressive relationship, can enhance teachers’ work enthusiasm and elevate teaching quality by fostering a positive campus environment and devising judicious teacher management strategies. Consequently, a favorable organizational climate can invigorate teachers’ enthusiasm and creativity, enhancing both work efficiency and teaching quality. Schools characterized by a robust collaborative climate, an open leadership style, and adaptable communication methods tend to cultivate higher levels of job satisfaction and a profound sense of achievement among teachers. In conclusion, the organizational climate of kindergartens had a positive predictive effect on the job satisfaction of kindergarten teachers (Hypothesis 1).

### 1.2 The mediating role of occupational stress

Occupational stress is characterized by adverse physiological or psychological reactions, including anger, irritability, loss, frustration, and anxiety, triggered when an individual confronts environmental stimuli and threats (Cooper and Cooper, 1983). Teachers’ professional stress is a series of physiological, psychological and behavioral responses that develop when their own abilities and available external resources cannot meet the demands of their daily work in their work climate (Zhou and Ning, 2020). The stress experienced by kindergarten teachers in teaching situations is a significant factor contributing to the turnover of kindergarten teachers and represents a crucial aspect influencing the job satisfaction of kindergarten teachers (Wang et al., 2015). Some scholars in China have found that the state of teachers’ professional survival is poor, and 92% of kindergarten teachers consider their work “very tiring” (Alatanbagen and Liu, 2014). Evidence shows that various factors are related to occupational stress, with high workload, long working hours, and high work intensity being proven as common high-risk factors (Basu et al., 2017). Prolonged and excessive occupational stress can cause dissatisfaction, passive laxity, high turnover and absenteeism among kindergarten teachers (Qin and Yan, 2007). High levels of work stress among kindergarten teachers can lead to burnout, and once burnout occurs, teachers’ job satisfaction declines (Ishibashi et al., 2022). Lower job satisfaction can lead to negative feelings among kindergarten teachers, who may withdraw from the profession altogether when negative feelings accumulate and reach a certain threshold (Grant et al., 2019). Inadequate welfare protection and unequal distribution of care and education in kindergartens contribute to the high level of professional stress among teachers (Xin et al., 2020). Occupational stress causes teachers to be negative and to question the status of their work, which directly affects the job satisfaction of kindergarten teachers (Smith, 2011). Therefore, occupational stress significantly influences teachers’ job satisfaction. In conclusion, the occupational stress of kindergarten teachers served as a mediator between kindergarten organizational climate and kindergarten teachers’ job satisfaction (Hypothesis 2).

### 1.3 The mediating role of emotional labor

Emotional labor refers to the camouflage and management of internal and external emotions by individuals to conform to organizational rules of expression (Hochschild, 1983). Teachers’ emotional labor is the psychological process through which educators’ endeavor to restrain or manage emotional feelings and expressive behaviors in alignment with professional norms and requirements within teaching and learning situations (Yin and Lee, 2012). Kindergarten teachers regulate their emotional feelings and expressions in line with specific requirements, rendering the care and education of young children a profession characterized by high emotional labor. The organizational climate in kindergartens directly or indirectly shapes the organizational behavior and values of kindergarten teachers, subsequently impacting their levels of physical and mental well-being (Suo, 2014). It is a key factor influencing teachers’ emotional labor. Kindergarten teachers experienced a more moderate organizational climate, and there was a notable correlation between director supportive behaviors in the kindergarten organizational climate and the emotional labor of kindergarten teachers (Wang, 2019). Relevant research has shown that teachers’ perceptions of the internal climate of their schools influence their choice of emotional labor strategies (Yao et al., 2015), Social support from leaders, colleagues, etc. in an organization can be effective in regulating anxiety, stress, etc. caused by emotional labor, and bring about a positive emotional experience for oneself (Wu, 2003). The traditional material-based management philosophy profoundly influences the externally pressurized and coercive management system of kindergartens, which seriously affects the quality of teachers’ emotional labor (Zhang, 2008). It has been shown that teachers’ emotional labor can have an impact on teachers’ job satisfaction. Teachers’ healthy and beneficial emotional labor strategies can significantly predict their teachers’ job satisfaction (Gu, 2022). For example, teachers who show more positive emotions (e.g., happiness) typically show higher job satisfaction (Brackett et al., 2012), kindergarten teachers are more likely to reduce their own job satisfaction when they frequently engage in surface-level play in their emotional labor efforts (Liu et al., 2018). As a result, the emotional labor of kindergarten teachers significantly affects teacher job satisfaction. Based on these, we hypothesized that emotional labor served as a mediator between kindergarten organizational climate and job satisfaction of kindergarten teachers (Hypothesis 3).

### 1.4 The chain mediating effect of occupational stress and emotional labor

Teacher occupational stress tends to lead to physical and psychological tension when teachers are confronted with threatening situations or adverse events in their profession (Liu, 2004). There is a general consensus in research that occupational stress has a positive effect on emotional labor as a whole, and that occupational stress and emotional labor are positively correlated. The amount of occupational stress among kindergarten teachers affects the choice of emotional labor styles; the greater the occupational stress, the greater the tendency to adopt surface behaviors and the less natural behaviors (Cai, 2022). Elevated stress levels result in heightened emotional exhaustion when teachers employ surface acting and proactive deep acting strategies. This impact extends to the overall well-being of teachers, affecting them both physically and mentally. Faced with occupational stress, kindergarten teachers often turn to emotional labor, involving the management and regulation of emotional expression through surface acting or proactive deep acting strategies. This type of emotional labor not only depletes teachers’ psychological resources but may also exacerbate emotional exhaustion, further contributing to adverse effects on their physical and mental well-being. Based on these theories and empirical findings, we assumed that the organizational climate of kindergartens might indirectly influence the job satisfaction of kindergarten teachers through their occupational stress and emotional labor (Hypothesis 4). Specifically, there was a chain-mediated effect, where kindergarten teachers’ occupational stress and emotional labor served as intermediate factors between kindergarten organizational climate and job satisfaction of kindergarten teachers.

## 2 Materials and methods

### 2.1 Participants

The research targeted kindergarten teachers across the nation, employing a convenient sampling method for a questionnaire survey. Electronic questionnaires were distributed through the online platform, Questionnaire Star. A total of 1,133 questionnaires were collected, and after excluding 42 invalid ones due to a short completion time and patterned responses, 1,091 valid questionnaires were retained, resulting in an effective rate of 96.3%. In the sample of kindergarten teachers, there were 24 males, constituting 2.2%, and 1,067 females, making up 97.8%. The majority held a Bachelor’s degree, accounting for 62%. Regarding teaching experience, 508 teachers had less than 5 years, making up 46.6%, 298 had 6–10 years (27.3%), 105 had 11–15 years (9.6%), and 180 had more than 15 years (16.5%). The largest percentage of kindergartens was located in cities, comprising 61.2%.

### 2.2 Measures

#### 2.2.1 Kindergarten organizational climate

This study utilized the ‘Kindergarten Organizational Climate Scale’ developed by Li et al. (2017) as the measurement tool. The scale, based on the OCDQ-RE scale by Hoy and Clover (1986), consists of 33 items categorized into six dimensions: principal supportive behavior, principal supervisory behavior, principal restrictive behavior, teacher alienation behavior, teacher intimacy behavior, and teacher dedication behavior. It employs a 5-point Likert scale ranging from 1 representing ‘very inconsistent’ to 5 representing ‘very consistent,’ with items 11 and 12 being reverse-scored. The higher the score on the scale, the higher the perceived organizational climate in the kindergarten. The study demonstrated good structural validity and reliability of the scale, with Cronbach’s α of 0.92 and Cronbach’s α coefficients for each dimension ranging from 0.87 to 0.91. Other indicators include x^2^/df = 2.51, RMSEA = 0.003, CFI = 0.93, NFI = 0.91, IFI = 0.91, GFI = 0.92, PNFI = 0.90, RMR = 0.04 (Zhao, 2023).

#### 2.2.2 Job satisfaction of kindergarten teachers

The Kindergarten Teacher Job Satisfaction Scale developed by Lu (2023) was employed in this study. The scale comprises a total of 18 items, including four dimensions: job stress, material rewards, work environment, and spiritual rewards. A Likert 5-point scoring method was used, ranging from 1 representing ‘strongly disagree’ to 5 representing ‘strongly agree.’ A higher total score indicates higher job satisfaction among kindergarten teachers. The research indicates that the scale has good reliability and validity, with a Cronbach’s α of 0.889 for the total scale and Cronbach’s α coefficients ranging from 0.781 to 0.903 for each dimension; *x*^2^/df = 2.710, RMSEA = 0.075, CFI = 0.932, NFI = 0.897, IFI = 0.932, GFI = 0.885 (Lu, 2023).

#### 2.2.3 Occupational stress of kindergarten teachers

Using the Professional Stress Questionnaire for Kindergarten Teachers developed by Alatanbagen and Liu (2014). The scale consists of 18 items, covering four dimensions: work difficulty and challenge, work responsibility and reward, work intensity, and management system and career development. It uses a Likert 5-point scoring system, ranging from 1 for ‘no pressure’ to 5 for ‘extreme pressure.’ A higher score indicates greater occupational stress among kindergarten teachers. Studies have shown that the Cronbach’s α coefficient for this scale is 0.96, and the Cronbach’s α coefficients for each dimension range from 0.79 to 0.92. The KMO value is 0.956, TLI = 0.94, IFI = 0.92, NFI = 0.91, RMR = 0.02, RMSEA = 0.10, indicating good fit and excellent reliability and validity of the scale (Wang, 2023).

#### 2.2.4 Emotional labor of kindergarten teachers

The Emotional Labor Scale for Kindergarten Teachers developed by Lu (2023) was used, which mainly referred to the Emotional Labor Strategies Scale developed by Diefendorff et al. (2005). It consists of three dimensions, namely, surface play, deep play, and naturalistic performance, with a total of 13 question items. The scale adopts the Likert 5-point measurement scoring, ranging from 1 representing ‘strongly disagree’ to 5 representing ‘strongly agree.’ Higher scores in each dimension reflect a stronger inclination. Research indicates good reliability and validity of the overall emotional labor scale for kindergarten teachers, meeting the study requirements. Cronbach’s alpha for the total scale was 0.757, and the Cronbach’s alpha coefficient values for the dimensions ranged from 0.779 to 0.817; KMO values were 0.849, *x*^2^/df = 2.028, RMSEA = 0.058, CFI = 0.953, NFI = 0.912, IFI = 0.953, and GFI = 0.939 (Lu, 2023). The model exhibits good fit as all indicators meet the requirements of measurement standards.

### 2.3 Data analysis

The data were statistically analyzed using SPSS 27.0 and PROCESS 4.2. Common method bias was initially assessed using Harman’s single-factor analysis. Descriptive statistics were then employed, and the reliability of the scales was evaluated through Cronbach’s Alpha coefficient. Pearson correlation coefficients were calculated to explore variable relationships. Finally, PROCESS (model 6) was utilized to investigate chain mediation relationships involving organizational climate, occupational stress, emotional labor, and job satisfaction.

## 3 Results

### 3.1 Common method bias inspection

As all data in this study were derived from self-reports of study participants, a common method bias effect may have been introduced (Zhou and Long, 2004). Therefore, the Harman’s One-factor Test was employed to analyze common method biases. The results indicate that there are 17 factors with eigenvalues greater than 1. The explained variance of the first factor is 27.893%, which is below the critical standard of 40%, suggesting the absence of a serious common method bias issue.

### 3.2 Descriptive statistics and correlation analyses of the research variables

Table 1 presents the mean, standard deviation, and correlation coefficient for each variable. Significant correlations were identified among kindergarten organizational climate, occupational stress, emotional labor, and job satisfaction of kindergarten teachers. Notably, kindergarten organizational climate demonstrated significant correlations with occupational stress, emotional labor, and job satisfaction. Occupational stress exhibited a significant correlation with emotional labor and job satisfaction, while emotional labor showed a significant positive correlation with job satisfaction. Furthermore, considering the varying degrees of correlation between demographic variables (education attainment, length of teaching experience, kindergarten level, and official staffing recommendations) and the four study variables, these demographic variables will be controlled for in subsequent tests.

**Table 1:** Correlation between the variables.

Variables	M ± SD	1	2	3	4	5	6	7	8
1. EA	2.380 ± 0.580	1							
2. TOTE	1.960 ± 1.105	−0.110^**^	1						
3. KL	2.080 ± 0.878	0.198^**^	−0.267^**^	1					
4. OSR	1.630 ± 0.482	0.386^**^	−0.323^**^	0.399^**^	1				
5. OC	3.733 ± 0.375	0.114^**^	0.000	0.007	0.105^**^	1			
6. OS	2.596 ± 0.880	−0.187^**^	0.119^**^	−0.138^**^	−0.157^**^	−0.181^**^	1		
7. EL	3.862 ± 0.566	0.031	0.128^**^	−0.051	−0.024	0.347^**^	0.124^**^	1	
8. JS	3.755 ± 0.405	−0.038	0.036	−0.048	−0.002	0.531^**^	−0.109^**^	0.313^**^	1

EA, education attainment; TOTE, length of teaching experience; KL, kindergarten level; OSR, official staffing recommendations; OC, organizational climate; OS, occupational stress; EL, emotional labor; JS, job satisfaction; ^**^p < 0.01, ^***^p < 0.001.

### 3.3 Kindergarten organizational climate and kindergarten teachers’ job satisfaction: a chain mediation effect test

For further investigation into the relationship between organizational climate, occupational stress, emotional labor, and job satisfaction, we conducted a mediation analysis using Model 6 from the SPSS macro PROCESS4.2 developed by Hayes. This analysis aimed to examine the mediating effects of occupational stress and emotional labor in the relationship between kindergarten organizational climate and job satisfaction among kindergarten teachers. The results of the analysis are presented in Table 2. Regression analyses revealed that organizational climate significantly predicted occupational stress and emotional labor (*β* = −0.2077, *p* < 0.001; *β* = 0.2248, *p* < 0.001); occupational stress significantly positively predicted emotional labor (*β* = 0.0861, *p* < 0.001); and emotional labor significantly positively predicted job satisfaction (*β* = 0.1554, *p* < 0.001). Thus, the chain-mediated effect of organizational Climate through occupational stress and emotional labor on job satisfaction is significant.

**Table 2:** Regression analysis of the relationship of variables in the model.

Regression equation		Overall fit index			Significance of regression coefficients		
Outcome	Predictor	*R*	R2	*F*	*β*	*SE*	*t*
OS	OC	0.2809	0.0789	18.5863	−0.1622	0.0377	−5.5125^***^
EL	OC	0.4114	0.1693	36.8173	0.3778	0.0169	13.3261^***^
	OS				0.1853	0.0134	6.4234^***^
JS	OC	0.5609	0.3146	71.0266	0.4765	0.0164	17.1449^***^
	OS				−0.0668	0.0123	−2.5014^*^
	EL				0.1570	0.0273	5.6867^***^

OC, organizational climate; OS, occupational stress; EL, emotional labor; JS, job satisfaction; ^*^p < 0.01, ^***^p < 0.001.

**Figure 1:**
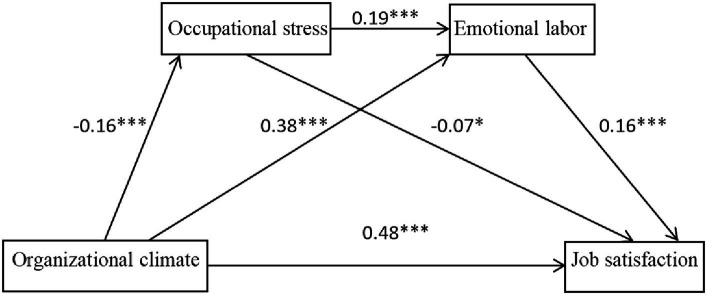
Chained intermediary model diagram. *p < 0.01, ***p < 0.001.

Following that, we utilized the Bootstrap method to examine and develop the chained mediation model. The number of draws was established at 5000, and the confidence interval was set at 95% to assess the significance of the total, direct, and mediating effects. If none of the path coefficients include zero within the 95% confidence interval, the statistic is considered statistically significant, signifying a significant mediation effect (Wen and Ye, 2014). The results of the path coefficient test and the model diagram are shown in Table 3 and Figure 1. Specifically, the compound multiple mediation effect of kindergarten organizational climate on teachers’ job satisfaction included three pathways of indirect effects of 0.0064 [95% CI (0.0008, 0.0129)], 0.0349 [95% CI (0.0216, 0.0488)] and −0.0028 [95% CI (−0.0046, −0.0014)]. The mediating effect sizes were 2.00, 10.93, and 0.89%, respectively; the total effect was 0.3194, the total indirect effect was 0.0386, accounting for 12.09% of the total predicted effect of kindergarten organizational climate on perceived satisfaction of kindergarten teachers. The 95% confidence intervals of the above indirect effects do not contain 0, indicating that they are statistically significant, and the coefficients of the paths are significant. Occupational stress and emotional labor exhibit significant chain mediation effects between kindergarten organizational climate and kindergarten teachers’ job satisfaction. The organizational climate not only significantly influences kindergarten teachers’ job satisfaction through the singular mediating role of occupational stress or emotional labor but also indirectly impacts kindergarten teachers’ job satisfaction through the chain mediating role of occupational stress, emotional labor, and occupational stress. Chain mediation significantly affects kindergarten teachers’ job satisfaction, and all three mediation paths are valid. In conclusion, the organizational climate of a kindergarten, as a comprehensive factor of the working environment, directly affects teachers’ work experiences and feelings. This influence is not direct and simple, but rather transmitted and adjusted through a series of mediating variables such as occupational stress and emotional labor. This means that the organizational climate of the kindergarten can affect teachers’ job satisfaction by influencing their occupational stress. In addition to occupational stress, emotional labor is also an important mediating variable. The organizational climate of the kindergarten can indirectly affect teachers’ job satisfaction by regulating their emotional labor state. At the same time, certain aspects of the kindergarten’s organizational climate may initially cause teachers to experience occupational stress, which in turn affects their emotional labor, and ultimately affects job satisfaction through occupational stress again. This complex chain mediation effect demonstrates the complexity of interactions among variables.

**Table 3:** Analysis of the mediating effect of kindergarten organizational climate and job satisfaction of kindergarten teachers.

	Effect	Boot SE	Boot LLCI	Boot ULCI	Effect ratio
Total effect	0.3194	0.0152	0.2895	0.3492	100%
Direct effect	0.2808	0.0164	0.2487	0.3129	87.91%
Total indirect effect	0.0386	0.0073	0.0242	0.0532	12.09%
OC-OS-JS	0.0064	0.0030	0.0008	0.0129	2.00%
OC-EL-JS	0.0349	0.0068	0.0216	0.0488	10.93%
OC-OS-EL-JS	−0.0028	0.0009	−0.0046	−0.0014	0.89%

OC, organizational climate; OS, occupational stress; EL, emotional labor; JS, job satisfaction.

## 4 Discussion

This study explores the mechanism by which kindergarten organizational climate influences kindergarten teachers’ job satisfaction, considering the mediating roles of occupational stress and emotional labor. The findings reveal significant correlations between organizational climate and job satisfaction, occupational stress, and emotional labor among kindergarten teachers. Organizational climate not only directly influences kindergarten teachers’ job satisfaction through occupational stress and emotional labor but also indirectly affects job satisfaction through the interconnected mediating effects of occupational stress and emotional labor.

First of all, the study investigated the direct impact of kindergarten organizational climate on the job satisfaction of kindergarten teachers. The findings indicate that a favorable organizational climate significantly and positively influences job satisfaction, aligning with existing research outcomes. The results are in line with social exchange theory, proposing that when employees perceive organizational support, respect, and care from their supervisors, they develop a heightened sense of responsibility toward the organization. This, in turn, fosters positive attitudes and behaviors, such as a commitment to staying in the organization for personal development (Liu et al., 2015). This suggests that a positive perception of the organizational climate by kindergarten teachers is associated with higher job satisfaction and a more effective contribution to the work of kindergarten teachers. In an environment characterized by mutual trust, cooperation, and support, kindergarten teachers can better explore their talents and potentials, feel acknowledged, and experience positive emotional outcomes. This positive cycle reinforces kindergarten educators’ self-satisfaction and overall job well-being, consequently enhancing their job satisfaction. Conversely, in an unfavorable organizational climate, kindergarten teachers may experience feelings of depression, unease, and dissatisfaction, leading to a loss of interest and motivation in their work. This can manifest in negative work attitudes and behaviors, including burnout and a tendency to leave their jobs. Therefore, establishing a positive organizational climate is crucial for improving the job satisfaction of kindergarten teachers. Kindergartens can achieve this by creating a professional development community for teachers, adopting open and innovative communication methods, and organizing team-building activities. Simultaneously, organizations should prioritize the psychological needs and emotional experiences of their employees. This approach can bolster employees’ sense of identity and belonging to the organization, consequently elevating their job satisfaction and fostering loyalty (Berberoglu, 2018). Based on these findings, in the future, the organizational climate of kindergartens can be leveraged as a practical tool to enhance teachers’ job satisfaction.

Secondly, the mediating role of occupational stresses among kindergarten teachers. It has been shown that kindergarten teachers, who deal with younger children and combine care and education at the same time, are under greater occupational stress than primary and secondary school teachers (Kyriacou and Sutcliffe, 1978). The study proposes that occupational stress serves as a partial mediator between kindergarten organizational climate and teachers’ job satisfaction. Firstly, organizational climate significantly and negatively predicts occupational stress. In other words, a supportive organizational environment reduces teachers’ perceived occupational stress. This reduction in stress is attributed to a positive organizational climate that fosters teachers’ professional development and value realization, effectively alleviating perceived stress. Second, the diminished occupational stress contributes to enhanced job satisfaction among kindergarten teachers. Studies in clinical psychology indicate that prolonged exposure to high stress levels leads to various negative consequences, affecting daily life, work, and learning. Kindergarten teachers, with organizational support, can regulate their occupational stress by believing in their ability to effectively address challenges in teaching and life through professional knowledge. This deepens their understanding of their work, ultimately boosting job satisfaction. Consequently, reducing occupational stress in kindergarten teachers facilitates the efficient development of teaching practice and mitigates the sharp decline in job satisfaction caused by a negative organizational climate.

Thirdly, the study reveals the partial mediating role of kindergarten teachers’ emotional labor. Specifically, it indicates that emotional labor partially mediates the association between kindergarten organizational climate and kindergarten teachers’ job satisfaction. In the field of sociology, the earliest studies of emotional labor indicated its value implication, that is, the status of emotional state at work is consistent with the status of physical and mental labor, and when employees display appropriate emotions in order to get paid for the work they have done, emotions are regarded as commodities with exchange value, and the commoditization of emotions has led to the emergence of emotional labor (Sun, 2013), emotional labor as an emotional response-based emotional regulation, such as the inability to truly change the inner feelings of employees, very easy to produce emotional disorders, which over time will result in the depletion of emotional resources, will increase the possibility of work fatigue and burnout, resulting in a reduction of work commitment, job satisfaction decrease (Grandey, 2000; Hülsheger and Schewe, 2011). The study aligns with the conclusion that positive emotional labor within a relaxed and democratic organizational climate fosters heightened job satisfaction among kindergarten teachers. Conversely, negative emotional labor in a restrictive and authoritarian organizational climate diminishes their job satisfaction. These findings indicate that kindergarten teachers can adeptly manage their emotions, showcasing positive and enthusiastic emotional states during positive emotional labor, resulting in increased job satisfaction. Conversely, negative emotional labor may result in a decline in job satisfaction. In instances where kindergarten teachers struggle to regulate their emotions effectively, displaying negative emotions such as excessive tension, anxiety, and irritability, they may experience fatigue and stress at work, ultimately impacting job satisfaction. This highlights the importance of recognizing the significance of emotional labor in the pursuit of pathways to enhance the job satisfaction of kindergarten teachers. In practical applications, efforts should be directed toward minimizing the adverse consequences of emotional labor and optimizing teachers’ emotional labor to yield positive outcomes and bolster job satisfaction.

Finally, this study establishes a chain mediation model involving kindergarten organizational climate, occupational stress, emotional labor, and job satisfaction of kindergarten teachers. It explores the interplay of these factors to comprehensively understand the influences on kindergarten teachers’ job satisfaction. The organizational climate in kindergartens acts as a top-down moderator affecting individual teachers’ job satisfaction. The research delves into the factors influencing kindergarten teachers’ job satisfaction and emphasizes the pivotal role of organizational climate in shaping their work experiences. The findings suggest that occupational stress and emotional labor of kindergarten teachers jointly mitigate the negative impact of a poor organizational climate on job satisfaction. Perceived support from the organizational climate enables teachers to enhance self-motivation, focus on self-competence development, and experience positive emotions, contributing to increased job satisfaction. This highlights the importance of fostering individual teacher development within the context of kindergarten organizational climate management, emphasizing professional competence and emotional well-being to alleviate occupational stress and promote positive emotional labor, ultimately enhancing job satisfaction.

## 5 Research limitations and future directions

In the context of this study, there are certain limitations that need clarification. Firstly, the number of male teachers in the selected sample is limited, and the subjects chosen may not be diverse enough, potentially reducing the generalizability of the research results. In the future, efforts could be made to expand the sample size and increase the proportion of male teachers to enrich the outcomes of the study. Secondly, this study adopted a cross-sectional research design, which is not conducive to explaining causal relationships among variables. In the future, a longitudinal research design or experimental study could be employed to validate the impact of occupational stress and emotional labor on the job satisfaction of kindergarten teachers. Finally, the mediation model indicates that occupational stress and emotional labor play a partial mediating role between organizational climate and job satisfaction, suggesting the existence of other mediating factors influencing job satisfaction. Future research could introduce additional mediating variables for further exploration, such as teaching efficacy and emotional exhaustion, to gain a more comprehensive understanding of the mechanism by which organizational climate affects job satisfaction.

## 6 Conclusion

In conclusion, the organizational climate in kindergarten is a crucial factor influencing the job satisfaction of preschool teachers. Additionally, current research results suggest that the relationship between organizational climate and job satisfaction may be mediated by occupational stress and emotional labor. These findings offer a deeper understanding of how to enhance the job satisfaction of preschool teachers, providing empirical evidence and valuable intervention insights for future research.

## Data availability statement

The raw data supporting the conclusions of this article will be made available by the authors, without undue reservation.

## Ethics statement

The studies involving human participants were reviewed and approved by China West Normal University. Written informed consent from the participants’ legal guardian/next of kin was not required to participate in this study in accordance with the national legislation and the institutional requirements. The studies were conducted in accordance with the local legislation and institutional requirements. The participants provided their written informed consent to participate in this study.

## Author contributions

WX: Resources, Writing – review & editing, Writing – original draft, Validation, Supervision, Software, Project administration, Methodology, Funding acquisition, Data curation, Conceptualization. YF: Methodology, Writing – review & editing, Writing – original draft, Visualization, Validation, Software, Data curation, Conceptualization. JB: Writing – review & editing, Methodology, Investigation, Formal analysis, Data curation. QZ: Writing – review & editing, Software, Resources, Methodology, Investigation. YW: Writing – review & editing, Visualization, Investigation, Formal analysis.
