# Supplementary material for: The relationship between organizational climate and job satisfaction of kindergarten teachers: a chain mediation model of occupational stress and emotional labor
Source: Front Psychol. 2024 May 28;15:1373892. doi: 10.3389/fpsyg.2024.1373892 (PMC11165699; doi:10.3389/fpsyg.2024.1373892)
Supplement: Supplementary file 3 [file Table_3.DOCX]

**<https://www.wjx.cn/vm/P4ozh1R.aspx>**

**（Questionnaire Star Link）**

**幼儿园组织气氛对幼儿教师工作满意度的影响调研**

尊敬的老师：

您好!感谢您在百忙之中填写该问卷。本问卷采用匿名方式，仅作为研究参考，并无对错之分，您的每个选择都具有十分重要的价值。

请您填前仔细阅读说明部分，再根据您的实际情况与感受完成。十分感谢您的帮助。

教师基本信息

1.您的性别是

A.男 B.女

2.您的婚姻状况是

A.未婚 B.已婚

3.您的最高学历是

A.研究生 B.本科 C.大专 D.中专(中师)

4.您的年龄是

A.30 岁以下 B.31-40 岁 C.41-50 岁 D.50 岁以上

5.您的教龄:

A.5 年以下 B.6-10 年 C.11-15 年 D.15 年以上

6.您的学校所在地是

A.城市 B.县镇 C.农村

7.您所在的幼儿园类别？

A.公办 B.民办

8.您所在幼儿园的等级是

A.省级示范园 B.市级示范园 C.其他

9.您所教的幼儿年龄段是

A.托班 B.小班 C.中班 D.大班

10.您所带班级的幼儿人数

A.20人以下 B.20-25人 C.26-30人 D.31-35人 E.35人以上

11.您是否在编？

A.是 B.否

12.您的月工资？

A.2000 元以下 B.2001-3000 元 C.3001-4000 元D.4001-5000 元 E.5001 元以上

幼儿园组织气氛量表

该项旨在测量幼儿园组织气氛，请您结合实际来填写。

1=非常不符 2=基本不符 3=一般 4=基本符合 5=非常符合

1.园长想方设法帮助教师解决困难的问题。

2.幼儿园每一项工作都需要经由园长指派。

3.教师之间互相尊重。

4.老教师能很快接纳新来的教师。

5.园长监督教师每一件事情。

6.教师积极参加各种与专业相关的在职进修。

7.教师之间来往密切，相互关心。

8.园长信赖教师的教学及处理问题的能力。

9.幼儿园的办事程序繁琐复杂。

10.教师努力克服或解决教学上的问题。

11.教师们不会彼此互相帮助与支持。

12.教师不关心幼儿园的措施。

13.园长重视改善教师们的福利待遇。

14.幼儿园里非教学事务太多，影响教师正常教学工作。

15.教师热衷于不断追求新知。

16.教师最亲密信赖的朋友是本园的同事。

17.教师们常谈论工作调动方面的事情。

18.当教师有好的表现时，园长会给予表扬。

19.教师能在工作中表现出高度的合作精神。

20.教师开会时，经常会上一致通过，会下议论纷纷。

21.园长给教师充分表达意见的机会。

22.园长亲自安排教师所担任的工作。

23.幼儿园中有些教师形成小团体，排斥异己。

24.园长对待教师一视同仁。

25.教师的非教学性工作非常繁重。

26.教师的教学缺乏沟通、合作与互助。

27.教师牢骚满腹，说其他人坏话。

28.园长巡视幼儿园班级的教学情况。

29.例行的管理工作干扰到教师正常的教学。

30.教师以幼儿园为荣，工作努力。

31.园长严密监督教师在幼儿园的有关活动。

32.教师参与学术研讨或进行教学研究。

33.园长检查教师的教学计划。

幼儿教师工作满意度量表

请您仔细阅读每一个题项，就您目前的工作状况而言，选择最符合您的一项。

1=非常不同意2=比较不同意3=一般4=比较同意 5=非常同意

1.我认为目前的工作安排对我具有挑战性，使我感到疲惫。

2.我认为工作任务量偏大，使我精疲力竭。

3.我认为工作时长偏长给我带来了疲劳感。

4.面对孩子时我需要时刻注意自己的言行，这会消耗大量的精力。

5.我觉得工作压力对我的精神和情绪产生了不良影响。

6.和我的付出相比，我对自己的工资待遇感到满意。

7.我对教师培训、进修的机会感到满意。

8.我认为职称评聘的机会是公平的，让我感到满意。

9.我对幼儿园的教学条件感到满意。

10.我对同事间的人际关系感到满意。

11.我对园长的管理方式感到满意。

12.看到孩子们的成长我感到很高兴。

13.我的付出能得到他人的肯定。

14.我认为家长能理解并支持教师工作。

1. 我觉得教学工作很有趣，和孩子们在一起很开心。
2. 和孩子们的相处是被滋养和疗愈的，我觉得很幸福。

17.我与孩子们的互动积极、和谐，使我感到身心愉悦。

18.我的付出能够得到孩子们的积极回应，我很享受这样的过程。

幼儿教师职业压力量表

请根据您的实际情况选择答案，注意不要遗漏题目。

1=没有压力 2=轻度压力 3=中度压力 4=较大压力 5=极大压力

1.说话太多太累。

2.工作没有成就感。

3.工作琐碎复杂、工作量大。

4.不清楚幼儿园远景和未来发展方向。

5.园内有不合理的规章制度。

6.家长过分溺爱自己的孩子。

7.社会希望自己集保姆、教师、研究者于一身。

8.付出与收入成反差。

9.进修、培训机会少或没有。

10.班级人数太多。

11.幼儿自理能力低下。

12.幼儿的问题太多且难以回答。

13.时常担心幼儿的安全问题。

14.对新的教学内容被动适应。

15.心理放松的方法和技巧了解的少。

16.现代教育技术和教育观念了解的少。

17.不能有效控制自己的紧张和焦虑。

18.不断学习才能胜任现在的工作。

幼儿教师情绪劳动量表

以下是您在幼儿园与幼儿互动过程中可能会产生的情绪体验，请您根据自身实际情况，选择最符合您的一项。

1=非常不符合 2=比较不符合 3=不确定 4=比较符合 5=非常符合

1. 面对孩子时，我会隐藏真实的感受，有意表现出亲切热情。
2. 当我心情不好时，我会转移注意力想些开心的事情，让自己以积极的情绪面对孩子。
3. 当孩子心情不好时，我会自然而然地展现出对他的关心和爱。
4. 在与孩子互动中，要表现适当的情绪，对我而言就如同表演一样。
5. 对孩子需要表现出来的情绪(如亲切、热情)。我只要适当展现一下就可以了。
6. 我会展现出工作时需要的情绪，但不会改变自己当时的内心感受。
7. 我展现给孩子的情绪是我的真实感受。
8. 面对孩子时，表现的情绪与我内心的感受不一样。
9. 我会尽力调整自己不好的情绪，由衷地以亲切、热情的态度对待孩子。
10. 我向孩子表达的情绪是自然流露的。
11. 面对孩子时，我所展现出的情绪是经过调整和修饰的。
12. 即使明知孩子无理，我仍能站在他的立场为其着想，诚心为其解决问题。
13. 如果需要向孩子表现出某种情绪(如热情等)，我会尽可能发自内心去感受。
